# An Unstructured Phylogeographic Pattern with Extensive Gene Flow in an Endemic Bird of South China: Collared Finchbill (*Spizixos semitorques*)

**DOI:** 10.3390/ijms12063635

**Published:** 2011-06-07

**Authors:** Bin Gao, Lijiang Yu, Yanhua Qu, Gang Song, Chuanyin Dai, Ruiying Zhang, Zuohua Yin, Kaifeng Wang, Xuebin Gao, Shou-Hsien Li, Fumin Lei

**Affiliations:** 1 Key Laboratory of Zoological Systematics and Evolution, Institute of Zoology, Chinese Academy of Sciences, Beijing 100101, China; E-Mails: quyh@ioz.ac.cn (Y.Q.); songgang@ioz.ac.cn (G.S.); daicy@ioz.ac.cn (C.D.); zhangry@ioz.ac.cn (R.Z.); yinzh@ioz.ac.cn (Z.Y.); 2 Graduate University of the Chinese Academy of Sciences, Beijing 100049, China; E-Mail: rahello1234@163.com (B.G.); 3 Shanghai Science & Technology Museum, Shanghai 200127, China; E-Mail: yulj1709@126.com (L.Y.); 4 Shaanxi Institute of Zoology, Xi’an 710032, China; E-Mails: wangkf@ms.xab.ac.cn (K.W.); gaoxb63@163.com (X.G.); 5 Department of Life Science, National Taiwan Normal University, Taipei 116, Taiwan; E-Mail: t43028@ntnu.edu.tw (S.H.L.)

**Keywords:** Collared Finchbill, East Asia, unglaciated region, phylogeography

## Abstract

Recent phylogeographical studies indicated that glacial oscillations played a key role on the phylogeographic pattern of extant species. As most studies have previously been carried out on heavily ice-covered regions, such as in European and North American regions, potential effects of climatic oscillations on species that are distributed on ice-free regions are less known. To address this, we investigated the phylogeographic pattern of an avian species endemic to South China, which was not glaciated during the Pleistocene glaciations. By using 2142 bp mitochondrial DNA, we identified 89 haplotypes defined by 39 polymorphic sites. A combination of high haplotype diversity (0.786–1.00) and low nucleotide diversity (0.00132–0.00252) was detected among geographic populations. Explicit genetic divergence was observed between *S. s. semitorques* and *S. s. cinereicapillus* but not detected among geographic populations of *S. s. semitorques.* Divergence time of the two subspecies was dated back to 87 Kyr which is congruent with the interglacial MIS 5. A weak phylogeographic structure due to strong gene flow among geographic populations was identified in this species, suggesting complex topography of South China has not formed barriers for this species.

## 1. Introduction

It is widely accepted that glacial oscillations during Pleistocene shaped the geographic distribution and genetic pattern of many plant and animal species on previously glaciated regions [1–3]. For species distributed in Europe and North America, their phylogeographic studies have shown congruent patterns. During glacial advance, these species receded to southern refugia. As ice sheets retracted, they experienced rapid expansions and recolonized northern latitudes after the Last Glacial Maximum (LGM) [3–5]. The repeated range expansions and retractions have considerably influenced their genetic diversity and population differentiations.

In contrast, the influence of recent climatic oscillations due to glacial cycles on the phylogeographic patterns of species in unglaciated regions, especially in East Asia, remain poorly understood [3,6,7]. Recently, comparative phylogeographic research in an unglaciated North American region has shown congruent isolated and differentiation patterns with those of species distributed on previously glaciated areas, despite the fact that in some cases they may be older (Pliocene) [8].

East Asia harbors one of the most diverse temperate fauna as it was never heavily covered by ice-sheets [9,10]. During Pleistocene, East Asia was a mosaic of mountains lower than 2000 m and characterized by a relatively mild Pleistocene climate [11–14]. The climatic stability and heterogeneous topology of this region potentially hosted microclimatic zones capable of supporting a variety of habitats over time [15]. Three previous avian studies of this region showed different evolutionary history compared with Europe and North America [14,16,17]. Thus, glacial refugia may have been available for East Asian species throughout their entire ranges, instead of being limited to several regions of Europe and North America [14]. Accordingly, we assume that the milder Pleistocene climate may have evoked less stress for East Asian species than European and North American species, and possibly these species have similar patterns.

To address this, we used two fragments of mitochondrial DNA to study the phylogeographical structure of a passerine species, the Collared Finchbill (*Spizixos semitorques*). The Collared Finchbill is endemic to south China, with two described subspecies: *S. s. semitorques* and *S. s. cinereicapillus. S. s. semitorques* cover a large area of South Mainland China, while *S. s. cinereicapillus* is only found in Taiwan. The Collared Finchbill distributes mainly in shrubbery, bamboo forests, and grassy areas on hilly and flat terrain below 1000 m, and its distribution is roughly between 20° N and 30° N in latitude, and 105° E and 120° E in longitude [18]. Understanding the genetic variation among populations and the past demographic processes sculpting the current population structure of *S. semitorques* might enrich our understandings about the role of climatic changes during the Pleistocene glaciations on the phylogeographical formation of birds endemic to South China.

## 2. Results and Discussion

### 2.1. Results

**Figure 1:**
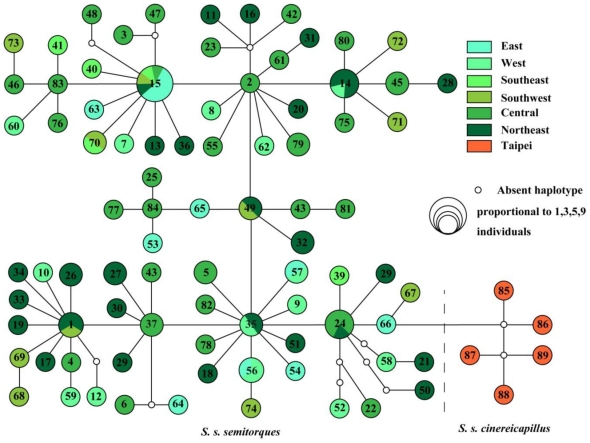
Haplotypes median-joining network for Collared Finchbill generated by NETWORK 4.6.

A combined length of 2142 bp mitochondrial genome, including Cytb (977 bp) and partial control region and tRNA-Glu (1165 bp) was obtained. Sequences aligned unambiguously have no gaps and there were no nonsense codons in protein-coding regions. Sixty-nine variable sites define 89 haplotypes. Most haplotypes are singletons, while there are only a few common haplotypes (10 see Attachment 1) shared by several geographical populations (Figure 1). The haplotype network depicts a compact topology and indicates no phylogeographic structure.

Neutrality test was conducted on geographic populations which contained at least five individuals. Tajima’s *D* values detected negative values for all populations, but none are significant (Table 1). Fu’s *Fs* statistics revealed significant negative values for SXFP (0.00) and GSWX (<0.01) populations. Fu and Li’s *D* tests showed negative values for SXFP (<0.05), HNYJ (<0.01) and GZGD (<0.01) populations. No population showed significant negative value in all three neutrality tests.

**Figure 2:**
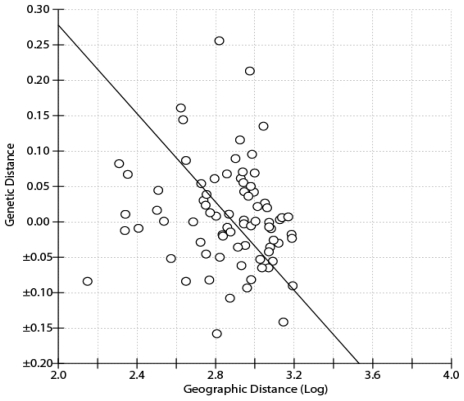
Data plot of the genetic distance (*F_st_*) *vs*. geographical distance (Log geographical distance in km) of the Mainland *Spizixos semitorques* populations, showing the RMA regression line (*R*^2^ = 0.0167).

AMOVA analysis show explicit divergence between two subspecies of Collared Finchbill (Table 2). We attempted to identify possible intraspecific genetic structure by defining several different ways of groupings in *S. s. semitorques*. However, AMOVA could not find a pattern with significant variation within any grouping. This indicates that there is no substantial genetic subdivision across subspecies on the mainland of China. Most of the variation is accounted for within-population variation, as could be expected given the large number of singletons. There is no correlation between *F_st_* genetic distance and geographic distance (Mantel test, Z = 118.0163, *r* = −0.1863, *p* = 0.9475) in *S. s. semitorques* (Figure 2).

**Figure 3:**
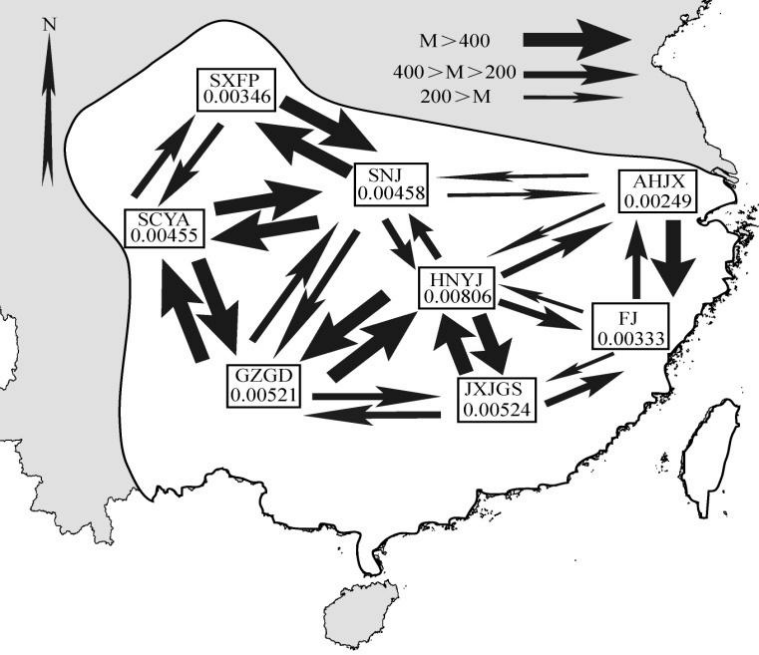
Effective population size and gene flow among populations estimated by LAMARC.

MDIV gave an average of 0.44, 0.311 and 20.39 for TMRCA, *t_pop_* and *θ* value respectively. Divergence time between two subspecies of Collared Finchbills was estimated to be about 87 Kyr. Meanwhile LAMARC estimated *θ* value for eight populations of *S. s. semitorques*. HNYJ population has a *θ* value of 0.00806 which was larger than any population around it. ANJX population got relatively lower *θ* value of 0.00249 and the remaining populations’ results range from 0.00333 to 0.00524. LAMARC also detected extensive gene flows among geographic populations of *S. s. semitorques* (Figure 3).

### 2.2. Discussion

Both local populations and the whole species of Collared Finchbill are characterized by high haplotype diversity and low nucleotide diversity. MtDNA markers detect an indistinctive genetic structure. These findings imply that the present populations of the *S. semitorques* seemed not to be affected by LGM and not segregated by topological complexity of South China. The results provide evidence of undiversified population structure.

Genetic variation is likely homogeneously distributed among populations on mainland China. The result suggested an undistinguished genetic structure of *S. s. semitorques* haplotypes bear strong signals of: an irregular haplotypes network, high haplotype diversity and low nucleotide diversity, and no significant negative value of Tajima’ *D* detected for all geographic populations.

Results of AMOVA also show that there is no population subdivision on a regional scale, and only a small amount of variation could be attributed to groups (~1.5%). The majority of the variations (99% variation is found within populations, Table 2) were found within *S. s. semitorques* populations, possibly as a result of strong gene flows among geographic populations. Mantel Test analyses do not indicate the pattern of isolation by distance among all sampling sites of *S. s. semitorques* in South China. This indicates there is no geographic barrier to segregate *S. s. semitorques*.

A pattern of high haplotype diversity and low nucleotide diversity is noted, which could be attributed to population expansion [19]. Given the harsh conditions during the Quaternary, there might have been numerous bottleneck events decreasing population size and genetic variability until such time as conditions became more favorable.

#### Demographic History

Although European and Asian continents cover similar latitude of Northern Hemisphere, they experienced different glacial histories [2,3,20]. Some compared phylogeographical researches indicated that Asian and European populations of the same species have obviously different genetic differentiation pattern [21–23]. This could reflect a different mechanism of species-specific responses to the glaciations. For example, many European species experienced post-glacial expansion due to the glaciations in the Quaternary, and expansion time roughly dated back to the retreat of the Last Maximun Glaciation (LGM) [2,3,20]. However, for many species distributes in Asia, their phylogeographic structures seemed not to be substantially affected by the LGM. Some extant species kept a stable population size during that time, but did not obviously expand with ice retreat after the LGM [14]. Three previous studies revealed intraspecific divergence and demographic history of some avian species in south China as a result of the Pleistocene climate change [14,16,17]. Mitochondrial data of *Leucodioptron canorum canorum* indicated the populations kept growing since penultimate ice age. This means complex topology of South China provided suitable habitats for *L. c. canorum.* Another example is *Alcippe morrisonia*, which widely distributes in South China and its ancestor dated back to 8 Myr. Geographical populations of *A. morrisonia* also kept stable or grew slightly during the LGM. *Bambusicola thoracica thoracica* showed a geographical structure among populations with little gene flow.

Unlike *Alcippe morrisonia* (Aves: Timaliidae), which is widespread in southern China and often flocks in the medium and understory of tropical rain forests and subtropical broadleaf evergreen forests [24,25], *S. semitorques* usually inhabits the canopy and edge of shrub land and has a larger body size [24] which might enable *S. semitorques* to have vagility and adaptability superior to *A. morrisonia*. The phylogeographical structure of *A. morrisonia* showed a deep lineage divergence while *S. semitorques* only have a shallow differentiation among geographical populations. Palynological and palaeoclimatic data suggest that the vegetation in East Asia in 35 Kyr ago similar to observed today might be ascribed to high precipitation, especially at middle or low latitudes [26–28]. The present results imply that during the cyclical oscillations of ice age, *S. semitorques* might stay relatively stable in certain areas of southern China.

East Asia has experienced several cyclical changes due to the sea level rising and falling. These sea level fluctuations started in the Early Miocene (24 Myr BP), and became more frequent after 10 Myr, and particularly during the Quaternary. Such changes of sea level led to occasional connection/disconnection between mainland and Taiwan. Divergence time of the two subspecies dated back to 87 Kyr BP while the TMRCA was estimated to 0.44 Myr BP. This result implied that the rising sea level did not effectively block the two subspecies for a long time until one of the warmest interglacial marine isotope stage 5 (MIS 5:130–74 Kyr) [29]. The sea level was even higher at that time than it presently is [30]; so it is probably that the Taiwan Strait virtually blocked gene flow between two subspecies of *S. semitorques* from then on.

## 3. Materials and Methods

### 3.1. Sample Collection, DNA Extraction, Amplification and Sequencing

**Figure 4:**
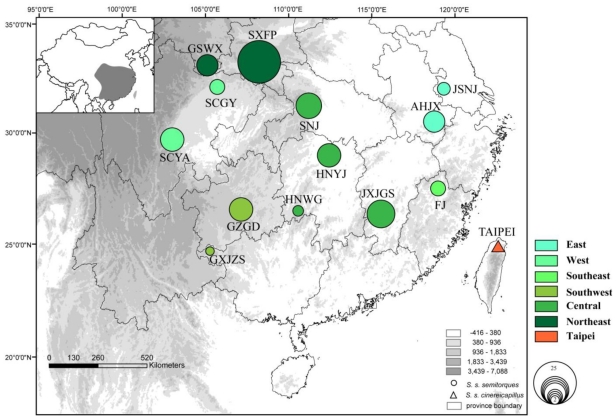
Sampling sites of *S. s. semitorques*: GSWX SXNS SCGY SCYA SNJ GZGD HNYJ JSNJ AHJX GXJZS JXJGS HNWG FJ; *S. s. cinereicapillus*: TAIPEI. Circles’ size is proportional to amount of each sampling site.

A total of 120 samples of Collared Finchbill were collected from 14 localities from 2003 to 2009, covering most of its distribution range in southern China (Table 3 and Figure 4). Liver/muscle samples were stored in 100% ethanol immediately after removal. Total genomic DNA was extracted from tissue samples using the DNeasy Blood and Tissue Kit (QIAGEN) following the manufacturer’s instructions. A partial cytochrome b (Cytb) gene of 977 (base pairs, bp) was amplified with the primer pair OSCL1 and OSCH2 [31]. The thermocycling program consisted of an initial denaturation at 94 °C for 5 min, followed by 40 cycles of 94 °C for 40 s, 53 °C for 40 s and 72 °C for 40 s, plus a final extension at 72 °C for 5 min. We used the primers L437 and H1248 [32] to amplify a fragment of 1165 bp from the central D-loop region to the end of the tRNA-Glu gene, including tRNA-Pro and ND6. The PCR thermal profile started with 94 °C for 3min followed by 30 cycles of: 94 °C for 30 s, 55 °C for 30 s, and 72 °C for 30 s. Sequencing using the H1248 primer was problematic, so a full sequence was obtained using two forward primers (16042: 5′-GTCACCAACTCCCAAAGC-3′ and 16520: 5′-ACCCAAAGCAAAACATAAACC-3′) under designed using Primer Premier 5.0 (Premier Biosoft International, Palo Alto, CA, USA). All individuals were sequenced in both directions (ABI377). The sequences were deposited to GenBank (cytb: JF509464-509583 and D-loop to tRNA-Glu: JF509584-509703).

### 3.2. Sequence Analysis

Fourteen sampling sites were identified for *S. s. semitorques* and one location for *S. s. cinereicapillus* (showed in Table 1). Sequences were aligned and edited using BioEdit 7.0 [33] and refined manually. DnaSP 5.0 [34] was used to calculate the number of segregating sites, haplotypes, haplotype diversity (h) [35], nucleotide diversity [36]. Genetic distance was computed using Arlequin 3.11 [37]. Tajima’s *D* [38] was used to examine the selective neutrality of mitochondrial fragments. Two additional neutrality tests, Fu and Li’s *D* [39] and Fu’s *F*_S_ [40] were used to detect departures from the mutation-drift equilibrium. F_S_ value tends to be negative if there is a significant excess of rare haplotypes, thus is often taken as evidence of recent demographic expansions or population bottlenecks [40]. All three tests were implemented in DnaSP 5.0 [34].

We used the combined sequences to reconstruct median-joining (MJ) network by program Network4.6 [41]. Mantel tests for isolation by distance (IBD) were performed using IBDWS [42]

MDIV [43] was employed to estimate divergence time and migration rate between two subspecies. Aligned sequence data from sample sites were used to estimate the parameters *H* (=2*N_e_l*), *M* (=*N_e_m* = number of migrants between populations per generation), and *T* (the divergence time between populations, where 1 time unit = *N*_e_ generations). Each analysis used an infinite-site model and 3000,000 generations of Markov chain Monte Carlo run and the first 500,000 generations was discarded as burn-in. Priors of maximum of M and T were set to 5 and 10 respectively. The divergence time between subspecies was estimated using the Formula *t*_divergence time_ = *T*_pop_ × (Theta/2 μk).

Molecular clock is widely used for the liable of sequence information. But the constancy of clocks have been controversial, particularly if they clash with estimates taken from more traditional sources such as the fossil record. Songbirds (Passeriformes) have a very limited fossil record in spite of making up more than one-half of the world’s 10,000 living species of birds. There has been no fossil reported for Pycnonotidae birds. Different species have different molecular clock due to life cycles and metabolic rates. So for a specific species, a useful clock should be close to calibrated relatives. We used conventional 2% [44–46] molecular clock as mutation rate and 1.7 years [47] as generation time for Collared Finchbill.

We estimated effective population size (Θ) and migration rate (*M*) in the assumption of both populations and migration are at equilibrium with the MCMC chain approach implemented in the program LAMARC 2.1.3 [48]. All analyses used a GTR model of evolution suggested by Model Test 3.7 [49]. Two simultaneous searches with heating scheme were run 10,000,000 steps with an initial burn-in of 1,000,000 steps. LAMARC assumed that a population has been growing at the same exponential rate for a long period of time. Therefore, the ability to detect population growth depends on the amount of time a population experienced growth. Positive values of g indicated a growing population, and negative values indicated a shrinking population from past to present. The maximum probability estimates (MPE) for population under growth is above zero on average. As g value tended to be inflated, 95% CI of g including zero suggested undistinguished deviation from constant population size. Analyses were repeated three times with different random number seeds to assess consistency. The population growth or decline was significant if the 95% confidence interval of g did not include zero. The posterior probability densities and ESSs for these estimated population parameters were calculated in Tracer 1.5 [50].

## 4. Conclusions

Our study showed undistinguished genetic structure among geographical populations of *S. semitorques*. Approximate random gene flow indicated there might be no distinctive geographic barriers for this species in south China during late Pleistocene, which differs from the other three species of previous studies. Shallow differentiation due to considerable gene flow and lack of barriers might lead to high haplotype diversity and relative low nucleotide diversity. The mtDNA data showed a short evolutionary history of *S. semitorques* and undistinguished effects of the LGM on its historical demography.

## Figures and Tables

**Table 1:** Neutrality test for geographic populations.

*S. s. semitorques*											*S. s. cinereicapillus*
Statistics	GSWX	SXFP	SCGY	SCYA	SNJ	JXJGS	HNYJ	GZGD	AHJX	FJ	TAIPEI
Tajima’s *D*	−0.933	−1.633	−0.109	−0.7064	−0.996	−0.694	−0.591	−0.691	−0.986	−1.199	−0.894
*P*	NS	NS	NS	NS	NS	NS	NS	NS	NS	NS	NS
Fu’s *Fs*	−4.843	−12.75	−1.283	−3.336	−2.752	−1.908	−5.719	−6.401	−2.764	−1.554	−1.633
*P*	<0.01	0.00	NS	NS	NS	NS	NS	NS	NS	NS	NS
Fu and Li’s *D*	−1.313	−2.803	−0.109	−0.640	−1.270	−0.798	−0.765	−1.360	−1.370	−1.199	−0.894
*P*	NS	< 0.05	NS	NS	NS	NS	< 0.01	< 0.01	NS	NS	NS

Note: Three neutrality test parameters and statistical p values were calculated by DnaSP 5.0.

**Table 2:** AMOVA analyses of Collared Finchbill.

Group	Among groups (AG)	Among populations within groups(AP)	Within populations (WP)	Percentage of variation
	FCT	FSC	FST	
2 Groups *S. s. semitorques* (mainland sites): *S. s. cinereicapillus* (TAIPEI)	0.5043	0.0098	0.5092	Among groups:50.44 Among populations within group: 0.49 Within populations: 49.08
6 Groups East(AHJX, JSNJ): West(SCYA, SCGY): Southeast(FJ): Southwest(GZGD, GXJZS): Central (JXJGS, HNYJ, HNWG, SNJ): Northwest(GSWX, SXFP)	0.0029	0.0067	0.0096	Among groups: 0.29 Among populations within group: 0.67 Within populations: 99.04

Note: For two groups, *S. s. semitorques* includes populations on mainland and *S. s. cinereicapillus* includes the population in Taiwan.

**Table 3:** Sampling information for Collared Finchbill.

Subspecies	Localities	Latitude	Longitude	Sample size	Haplotype numbers	Nucleotide diversity (π)	Haplotype diversity (Hd)
*S. s. semitorques*	GSWX	32°56′37.74″	104°41′0.16″	9	1/13/14/15/16/17/18/19/21	0.00236	1.000
	SXFP	33°11′24.0″	108°12′0.0″	25	1/11/14/20/24/26/27/28/29/30/31/32/33/34/35/36/38/49/50/51	0.00231	0.980
	SCGY	32°25′44.51″	105°54′4.61″	5	7/8/9/10/12	0.00243	1.000
	SCYA	29°35′24.0″	102°35′24.0″	10	14/35/52/56/57/58/59/60/62	0.00252	0.978
	SNJ	31°24′35.6″	110°33′18.5″	11	2/3/4/5/6/37/45/55/61	0.00236	0.964
	HNYJ	28°55′53.0″	112°17′39.7″	10	22/24/25/42/43/44/46/47/48	0.00240	0.978
	HNWG	26°25′48.0″	110°22′12.0″	3	15/23/24	0.00187	1.000
	GZGD	26°35′4.96″	107°14′3.46″	10	1/15/49/68/69/70/71/72/73/	74 0.00245	1.000
	GXJZS	24°39′59.36″	104°52′17.8″	2	37/67	0.00187	1.000
	JXJGS	26°32′38.44″	114°8′50.45″	12	24/75/76/77/78/79/80/81/82/83/84	0.00195	0.985
	JSNJ	32°1′48.00″	118°27′36.0″	4	53/54/56/57	0.00210	1.000
	AHJX	30°4′15.12″	118°35′44.45″	9	15/63/64/65/66	0.00132	0.786
	FJ	27°19′54.86″	118°7′13.85″	5	15/39/40/41/70	0.00205	1.000
*S. s. cinereicapillus*	TAIPEI	24°54′56.56″	121°40′26.17″	5	85/86/87/88/89	0.00196	1.000

Note: Haplotype numbers are also used in network analysis and underlines denote shared haplotype.

## References

[1] Avise J (2000). Phylogeography: The History and Formation of Species.

[2] Hewitt G (2000). The genetic legacy of the Quaternary ice ages. Nature.

[3] Hewitt G (2004). Genetic consequences of climatic oscillations in the Quaternary. Phil. Trans. Roy. Soc. London B.

[4] Taberlet P, Fumagalli L, Wust-Saucy AG, Cosson JF (1998). Comparative phylogeography and postglacial colonization routes in Europe. Mol. Ecol.

[5] Lessa EP, Cook JA, Patton JL (2003). Genetic footprints of demographic expansion in North America, but not Amazonia, during the Late Quaternary. Proc. Nat. Acad. Sci. USA.

[6] Zhang Y, Ge S (2007). Molecular evolution study in China: Progress and future promise. Phil. Trans. Roy. Soc. London B.

[7] Qiu YX, Fu CX, Comes HP (2011). Plant molecular phylogeography in China and adjacent regions: Tracing the genetic imprints of Quaternary climate and environmental change in the world’s most diverse temperate flora. Mol. Phylogenet. Evol.

[8] Soltis DE, Morris AB, McLachlan JS, Manos PS, Soltis PS (2006). Comparative phylogeography of unglaciated eastern North America. Mol. Ecol.

[9] Shi Y (1986). Quaternary glaciation in China. Quatern. Sci. Rev.

[10] Liu K (1988). Quaternary history of the temperate forests of China. Quatern. Sci. Rev.

[11] Weaver A, Eby M, Fanning A, Wiebe E (1998). Simulated influence of carbon dioxide, orbital forcing and ice sheets on the climate of the last glacial maximum. Nature.

[12] Pinot S, Ramstein G, Harrison S, Prentice I, Guiot J, Stute M, Joussaume S (1999). Tropical paleoclimates at the last glacial maximum: comparison of Paleoclimate Modeling Intercomparison Project (PMIP) simulations and paleodata. Clim. Dynam.

[13] Ju L, Wang H, Jiang D (2007). Simulation of the last glacial maximum climate over East Asia with a regional climate model nested in a general circulation model. Palaeogeogr. Palaeoclimatol.

[14] Li S, Yeung C (2009). Sailing through the Late Pleistocene: unusual historical demography of an East Asian endemic, the Chinese Hwamei (*Leucodioptron canorum canorum*), during the last glacial period. Mol. Ecol.

[15] Qian H, Ricklefs RE (2000). Large-scale processes and the Asian bias in species diversity of temperate plants. Nature.

[16] Song G, Qu Y, Yin Z, Li S, Liu N, Lei F (2009). Phylogeography of the *Alcippe morrisonia* (Aves: Timaliidae): Long population history beyond late Pleistocene glaciations. BMC Evol. Biol.

[17] Huang Z, Liu N, Liang W, Zhang Y, Liao X, Ruan L, Yang Z (2010). Phylogeography of Chinese bamboo partridge, *Bambusicola thoracica thoracica* (Aves: Galliformes) in south China: Inference from mitochondrial DNA control-region sequences. Mol. Phylogenet. Evol.

[18] MacKinnon J, Phillipps K, He F (2000). A Field Guide to the Birds of China.

[19] Grant W, Bowen B (1998). Shallow population histories in deep evolutionary lineages of marine fishes: insights from sardines and anchovies and lessons for conservation. J. Hered.

[20] Hewitt G (1996). Some genetic consequences of ice ages, and their role, in divergence and speciation. Biol. J. Linn. Soc.

[21] Kryukov A, Iwasa M, Kakizawa R, Suzuki H, Pinsker W, Haring E (2004). Synchronic east-west divergence in azure-winged magpies (*Cyanopica cyanus*) and magpies (*Pica pica*)*. J. Zool. Syst. Evol. Res.

[22] Zink RM, Drovetski SV, Questiau S, Fadeev IV, Nesterov EV, Westberg MC, Rohwer S (2003). Recent evolutionary history of the bluethroat (*Luscinia svecica*) across Eurasia. Mol. Ecol.

[23] Pavlova A, Zink RM, Drovetski SV, Red’kin Y, Rohwer S (2003). Phylogeographic patterns in *Motacilla flava* and *Motacilla citreola*: species limits and population history. Auk.

[24] Zou F, Chen G (2004). A study of understory bird communities in tropical mountain rain forest of Jianfengling, Hainan Island, China. Acta Ecol. Sinica.

[25] Taiwanica AZ (1998). Diet analysis of the gray-cheeked Fulvetta (*Alcippe morrisonia*) at Fushan Experimental Forest in Taiwan. Acta Zool. Taiwan.

[26] Yuan D, Cheng H, Edwards R, Dykoski C, Kelly M, Zhang M, Qing J, Lin Y, Wu J (2004). Timing, duration, and transitions of the last interglacial Asian monsoon. Science.

[27] Kelly M, Edwards R, Cheng H, Yuan D, Cai Y, Zhang M, Lin Y, An Z (2006). High resolution characterization the Asian Monsoon between 146,000 and 99,000 years BP from Dongge Cave, China and global correlation of events surrounding Termination II. Palaeogeogr. Palaeoclimatol.

[28] Yu G, Gui F, Shi Y, Zheng Y (2007). Late marine isotope stage 3 palaeoclimate for East Asia: A data-model comparison. Palaeogeogr. Palaeoclimatol.

[29] Zhao B, Wang Z, Chen J, Chen Z (2008). Marine sediment records and relative sea level change during late Pleistocene in the Changjiang delta area and adjacent continental shelf. Quatern. Int.

[30] Hearty PJ, Hollin JT, Neumann AC, O’Leary MJ, McCulloch M (2007). Global sea-level fluctuations during the Last Interglaciation (MIS 5e). Quatern. Sci. Rev.

[31] Qu Y, Lei F (2009). Comparative phylogeography of two endemic birds of the Tibetan plateau, the white-rumped snow finch (*Onychostruthus taczanowskii*) and the Hume’s ground tit (*Pseudopodoces humilis*). Mol. Phylogenet. Evol.

[32] Tarr C (1995). Primers for amplification and determination of mitochondrial control-region sequences in oscine passerines. Mol. Ecol.

[33] Hall T (1999). BioEdit: A User-Friendly Biological Sequence Alignment Editor and Analysis Program for Windows 95/98/NT. Nucleic Acids Symp.

[34] Librado P, Rozas J (2009). DnaSP version 5.0: A software for comprehensive analysis of DNA polymorphism data. Bioinformatics.

[35] Nei M (1987). Molecular Evolutionary Genetics.

[36] Nei M, Tajima F (1981). DNA polymorphism detectable by restriction endonucleases. Genetics.

[37] Excoffier L, Laval G, Schneider S (2005). Arlequin (version 3.0): An integrated software package for population genetics data analysis. Evol. Bioinform.

[38] Tajima F (1989). Statistical method for testing the neutral mutation hypothesis by DNA polymorphism. Genetics.

[39] Fu Y, Li W (1993). Statistical tests of neutrality of mutations. Genetics.

[40] Fu YX (1997). Statistical tests of neutrality of mutations against population growth, hitchhiking and background selection. Genetics.

[41] Bandelt H, Forster P, Röhl A (1999). Median-joining networks for inferring intraspecific phylogenies. Mol. Biol. Evol.

[42] Jensen J, Bohonak A, Kelley S (2005). Isolation by distance, web service. BMC Genet.

[43] Nielsen R, Wakeley J (2001). Distinguishing migration from isolation: A Markov chain Monte Carlo approach. Genetics.

[44] Weir J, Schluter D (2008). Calibrating the avian molecular clock. Mol. Ecol.

[45] Weir JT (2006). Divergent timing and patterns of species accumulation in lowland and highland neotropical birds. Evolution.

[46] Lovette IJ (2004). Mitochondrial dating and mixed support for the “2% rule” in birds. Auk.

[47] Sæther BE, Lande R, Engen S, Weimerskirch H, Lillegard M, Altwegg R, Becker PH, Bregnballe T, Brommer JE, McCleery RH, Merilä J, Nyholm E, Rendell W, Tryjanowski P (2005). Generation time and temporal scaling of bird population dynamics. Nature.

[48] Kuhner M (2006). LAMARC 2.0: Maximum likelihood and Bayesian estimation of population parameters. Bioinformatics.

[49] Posada DC, Crandall KA (1998). MODELTEST: Testing the model of DNA substitution. Bioinformatics.

[50] Rambaut A, Drummond AJ (2007). Tracer v1.5.

